# Myopathy in the York Platelet Syndrome: An Underrecognized Complication

**DOI:** 10.1155/2018/5130143

**Published:** 2018-08-12

**Authors:** Joy Roman, Michael I. Palmer, Cheryl A. Palmer, Nicholas E. Johnson, Russell J. Butterfield

**Affiliations:** ^1^Department of Pathology, University of Utah, Salt Lake City, UT, USA; ^2^Department of Neurology, University of Utah, Salt Lake City, UT, USA; ^3^Department of Pediatrics, University of Utah, Salt Lake City, UT, USA

## Abstract

York Platelet Syndrome (YPS) is a calcium channelopathy caused by gain of function in STIM1, a gene which acts as a calcium sensor. It is characterized by platelet abnormalities and muscle weakness. Medical literature emphasizes the hematologic aspects of the cases with few data of the neuromuscular and neuropathologic evaluation. We present a patient with YPS whose myopathy was the most prominent aspect. She presented around 2 years of age with proximal weakness and easy bruisability. YPS was diagnosed in the infant at 16 months of age at the National Institutes of Health. Muscle biopsy demonstrated a severe chronic myopathy. Rimmed vacuoles and tubular aggregates were noted. Although YPS is rare, the combination of a congenital myopathy with thrombocytopenia may facilitate the diagnosis and enable further insights into the disease.

## 1. Introduction

York Platelet Syndrome (YPS) is a calcium channelopathy caused by gain of function in STIM1, a gene which acts as a calcium sensor. It is characterized by thrombocytopenia and deficient platelet calcium storage in delta granules. It presents overlapping symptoms with Stormorken syndrome. In 2015, Rabbind et al. suggested that Stormorken and YPS may be two different names for the same clinical condition [1]. The syndrome is rare, with less than 20 cases reported. Prior reports emphasize the platelet abnormalities in the syndrome, with minimal references to the myopathy and muscle biopsy abnormalities that occur in conjunction with the platelet defects [2]. We wish to further characterize this myopathy clinically and pathologically in a patient with YPS harboring a de novo activating mutation in STIM1, whose platelet abnormalities have been previously described as part of a case series [3, 4], as well as provide a review of the pathophysiology of this disease.

## 2. Case Description

This female infant was a term vaginal delivery to a G2P2002 mother with no complications during pregnancy. She had a normal newborn period aside from a small right parietal scalp hemangioma noted at birth. By 10 months of age, bruising on the lower extremities was noted in routine check-up notes. Hemoglobin and hematocrit at 11 months of age were slightly low (10.1 g/dL and 31.3%, respectively). At approximately 15 months of age, the infant's parents expressed concern over easy bruising. At approximately 16 months of age, a complete blood count (CBC) showed a platelet count of 104,000/uL, increased red cell distribution width (RDW, 17.1%), anisocytosis, and microcytosis. Around 20 months of age, the infant was brought to the emergency department for epistaxis. CBC was not performed at that time due to cessation of bleeding. By 24 months, her parents expressed concern over frequent tripping. At 25 months, her parents requested to obtain repeat CBC due to continued concerns regarding bruising and epistaxis. CBC at this time showed a platelet count of 119,000/uL, an increased RDW (18.2%), anisocytosis, and large platelets. Von Willebrand disease testing was negative. Platelet function testing was performed; the results were abnormal (prolonged with COL/EPI >300 sec, COL/ADP 142 sec). Initial diagnosis of Gray Platelet Syndrome was suggested. GATA-1 testing was normal. The child's family temporarily relocated to another state at this time. Hematology, orthopedics, and neurology evaluation were performed around the age of 3 years. Hematology suspected a combined alpha and dense granule platelet defect. Evaluation by orthopedics regarding gait abnormalities found an elevated CK (668 u/L), a positive Gower's sign, and the inability to climb stairs. Imaging procedures ruled out hip dysplasia and scoliosis. Neurologic examination identified Gower's sign as seen in prior examination, as well as a Trendelenburg gait, inability to run (describing it as more of a “fast walk”), wide base, normal range of motion, decreased tone with general weakness (proximal greater than distal), and normal sensory and reflex responses. The family returned to Utah shortly after this and resumed care. The parents enrolled the child at 3.5 years in an investigational platelet study at the National Institutes of Health (NIH). Blood samples of both the child and her mother were evaluated (the mother's peripheral smear had initially been thought to have a similar appearance to the child's, but this was later dismissed). The NIH identified the platelet abnormalities as the same seen in the two initial patients from the York family, for whom York Platelet Syndrome is named. They also noted the shared features of a mitochondrial myopathy [4]. Following this diagnosis, the patient was referred to the Unknown Diseases Program at the NIH. The NIH eventually identified a de novo STIM1 (c.343A>T) mutation responsible for a gain of function of the STIM1 protein and a CRAC channelopathy [3]. This mutation changes an isoleucine to a phenylalanine within the ncEF hand (I115F). Though not a mutation in the calcium-binding EFh of the STIM1 protein, this mutation is thought to destabilize the ncEFh/cEFh-SAM interaction, converting the N-terminus of STIM into an active conformation, regardless of calcium binding [5].

Nerve conduction studies and electromyography done on the patient through the NIH were consistent with a primary muscle disorder with membrane irritability [3].

**Figure 1:**
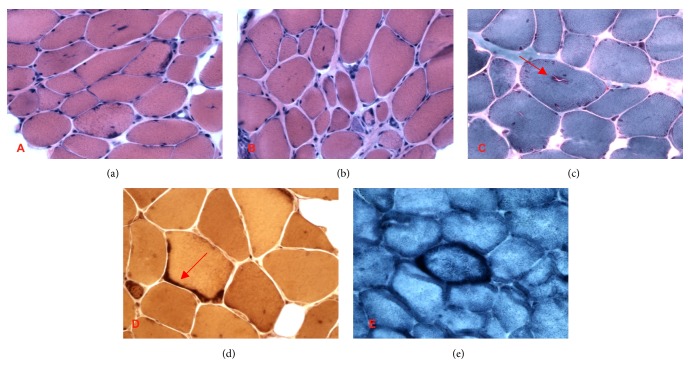
(a) Frozen section, H&E stain, showed mild fiber size variation and increased central nuclei. (b) Frozen section, H&E stain, demonstrated chronic fibrosis, fiber size variation, occasional central nuclei, and scattered degenerating and regenerating fibers. (c) Frozen section, Gomori trichrome stain, revealed occasional rimmed vacuoles (arrow). (d) Frozen section, nonspecific esterase (NSE) stain, also revealed occasional tubular aggregates (arrow). (e) Frozen section, NADH-tetrazolium reductase (NADH-TR) stain, revealed occasional tubular aggregates.

**Figure 2:**
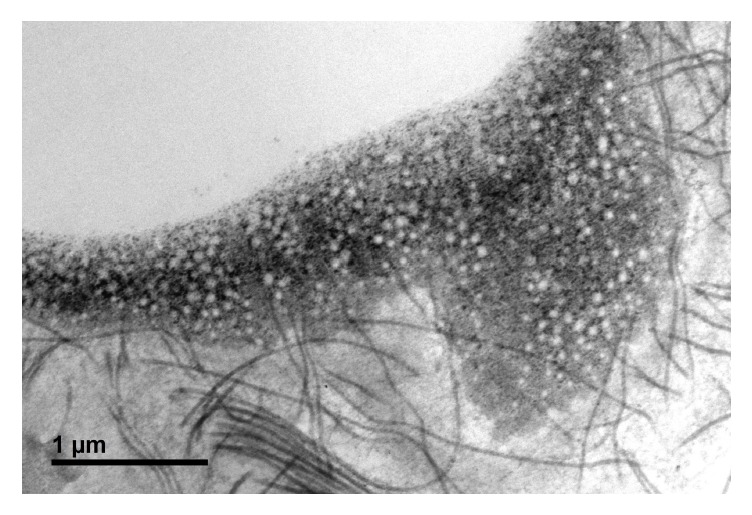
Electron microscopy showed tubular aggregates (x 6000.).

A muscle biopsy was performed at 9 years of age to evaluate the histologic features of the myopathy (Figure 1) and showed a tubular aggregate myopathy with rimmed vacuoles, moderate muscle fiber size variation, frequent atrophic and hypertrophic fibers, rare regenerating fibers, increased internal nuclei, no inflammation, and a mild type I fiber predominance. Areas of muscle tissue were replaced by adipose. Electron microscopic examination confirmed the presence of tubular aggregates (Figure 2).

## 3. Discussion

This case points out a novel pathological finding seen in YPS, tubular aggregates on muscle biopsy. Other patients with YPS have previously had myopathic features described, but there is limited information regarding the muscle pathology. Tubular aggregates have, to our knowledge, not yet been described in YPS patients, though this myopathy-related finding is unsurprising given that both mutations identified in YPS are seen in other syndromes that involve myopathy with tubular aggregates. Bohm et al. [6] characterized several tubular aggregate myopathy (TAM) patients with histologic findings of predominantly type II fiber atrophy with tubular aggregates. They identified STIM1 missense mutations in affected individuals, all of which were located in either the ncEF or cEF hands. Bohm et al. transfected STIM1-YFP constructs into C2C12 myoblasts harboring various STIM1 mutations identified in their TAM patients. This resulted in STIM1 clustering independent of the presence of thapsigargin (a SERCA inhibitor used to deplete SR calcium stores, eliciting STIM1 activation). Using muscle biopsies from these patients, they demonstrated higher basal cytoplasmic calcium levels and higher cytoplasmic calcium influx. These findings supported the idea of gain of function STIM1 mutations producing TAM. Although neither YPS nor Stormorken syndrome was mentioned in Bohm's report [6], there is clear overlap among these syndromes. Clinically, the combination of a platelet disorder and myopathy is a unique feature of YPS and should be considered in children who present with congenital myopathic features. Moreover, children presenting with a tubular aggregate myopathy should be evaluated for a STIM1 mutation. It is possible that, in additional patients with STIM1 mutations identified, individuals with an isolated tubular aggregate myopathy may have concurrent platelet dysfunction that is poorly characterized.

## 4. Conclusions

Though it has not previously been described specifically in YPS patients, the presence of tubular aggregates is not surprising, given the genotypic and phenotypic overlap with other STIM1-mutation related syndromes. The medical literature on YPS focuses on the platelet abnormalities, with less emphasis on the neuromuscular and neuropathologic aspects of the cases. Recognition of this presentation and the biopsy findings may enable clinicians to diagnose other patients with this unusual syndrome.

## References

[1] Singh A. R., Morin G., Rochette J. (2015). Stormorken syndrome or York platelet syndrome: A clinician's dilemma. *Molecular Genetics and Metabolism Reports*.

[2] (2018). *Online Mendelian Inheritance in Man [OMIM^®^]*.

[3] Markello T., Chen D., Kwan J. Y. (2015). York platelet syndrome is a CRAC channelopathy due to gain-of-function mutations in STIM1. *Molecular Genetics and Metabolism*.

[4] White J. G., Gunay-Aygun M. (2011). The York Platelet Syndrome: A third case. *Platelets*.

[5] Stathopulos P. B., Zheng L., Li G.-Y., Plevin M. J., Ikura M. (2008). Structural and Mechanistic Insights into STIM1-Mediated Initiation of Store-Operated Calcium Entry. *Cell*.

[6] Böhm J., Chevessier F., De Paula A. M. (2013). Constitutive activation of the calcium sensor STIM1 causes tubular-aggregate myopathy. *American Journal of Human Genetics*.

